# “Everyone can take photos.” Feasibility and relative validity of phone photography-based assessment of children’s diets – a mixed methods study

**DOI:** 10.1186/s12937-020-00558-4

**Published:** 2020-05-27

**Authors:** Åsa Norman, Karin Kjellenberg, Diana Torres Aréchiga, Marie Löf, Emma Patterson

**Affiliations:** 1 Department of Global Public Health, Karolinska Institutet, 171 77 Stockholm, Sweden; 2 Department of Biosciences and Nutrition, Karolinska Institutet, 141 83 Huddinge, Sweden; 3 Department of Health, Medicine and Caring Sciences, Linköping University, 581 83 Linköping, Sweden; 4 Centre for Epidemiology and Community Medicine, Stockholm Health Care Services, Region Stockholm, 104 31 Stockholm, Sweden

**Keywords:** Dietary assessment, Socioeconomic position, Validation, Acceptability, Mobile phone, Ecological momentary assessment

## Abstract

**Background:**

Dietary assessment methods that are user-friendly, simple, yet valid are of interest to both researchers and participants, particularly for use in disadvantaged settings, where language barriers and low levels of education are often present. We tested if parents taking photos of what children ate, using mobile phones, would be a feasible, acceptable method that could still provide information with adequate relative validity.

**Methods:**

We used a mixed-methods design, with parents of 21 5- to 7-year-olds from disadvantaged areas in Sweden. Parents reported all dietary intake, during non-school hours, on three days (two weekdays) using a photo method (PM). The PM consisted of simple instructions and a fiduciary card, but no training, equipment or software. Text messages could be sent if necessary. As a reference method, parents completed three 24-h recalls (24HRs) with an interviewer each following day. The next week, parents completed a 9-item semi-FFQ regarding the preceding week. The outcomes were intakes (in dl) of 9 food groups, categorised as fruits and vegetables, energy-dense sweet/salty foods, and sweet drinks. Agreement with the reference 24HRs was assessed using correlations, median differences and Bland-Altman plots. Parents completed an open-ended questionnaire on barriers and facilitators. Data collectors provided complementary information. Qualitative data was analysed using qualitative manifest analysis.

**Results:**

Nineteen parents (90%) provided complete data. The majority (*n* = 13) spoke Swedish as a second language, few (*n* = 4) were proficient. Compared to 24HRs, intakes measured by PM correlated well for all categories (Spearman’s rho = 0.609–0.845). However, intakes were underreported, significantly so for fruits and vegetables; Bland-Altman plots indicated that the underestimation was fairly constant across intake levels. When the FFQ was compared to the 24HRs, parameters of agreement were generally inferior than for the PM. Parents found the PM a positive experience, primarily facilitated by its simplicity and familiarity. Barriers, mainly related to time and logistics, can inform further methodological refinements.

**Conclusions:**

The PM was an acceptable and feasible way to measure children’s diet outside of school hours in this population of parents from disadvantaged areas. While the absolute validity should be evaluated further, this relatively simple method has potential for assessing intakes of well-defined foods at group level.

## Background

Measuring dietary intakes accurately remains a significant challenge for the field of nutritional epidemiology. The traditional and still dominant methods are questionnaires, written records and recalls, each with their advantages and disadvantages [1]. Questionnaires are cognitively burdensome, rely on long-term memory and as they are usually ‘closed’ or fixed, must be adapted for each context, to reflect foods common in each target population. Written records are open-ended, so allow for all types of foods to be reported, but impose a high burden, requiring very motivated participants, and often lead to unintended changes in behaviour. Recalls also rely on memory, albeit shorter term, and, depending on whether they are interviewer-mediated or self-administered, create a burden for participants and/or researchers.

There have long been calls for more innovative dietary assessment methods which, through the use of technology, can potentially reduce misreporting and recall biases and errors [2, 3]. Progress has been made in this field and many methods have been developed, categorised in a review in 2012 by Illner et al. as mobile phone-based, personal digital-assistant based, interactive computer-based, web-based, camera- and tape-recorder-based, or scan- and sensor-based [4]. However, issues and costs related to software development, data protection/storage, and equipment can be prohibitive for both researchers with limited resources and/or their intended participants. This is particularly the case for image-based methods where data volumes can be substantial [5–9].

A positive feature of technology-based methods is they are often more acceptable to participants than the traditional methods mentioned above [4, 5, 10]. When the acceptability and feasibility of new methods is evaluated it is often done quantitively, yet qualitative evaluations can provide important in-depth information regarding usefulness and perceptions of the method among intended users, further informing method development and refinement.

The potential advantages of newer methods, such as their increased acceptability and lower participant burden, can be particularly important when it comes to reaching certain vulnerable or disadvantaged subgroups of the population. Groups who may have lower levels of education, have difficulties with the local language, and - of relevance to dietary assessment - eat foods from another food culture. Disadvantaged groups often have greater health needs yet tend to participate less frequently in surveys and research in general [11]. Image-based methods for dietary assessment are particularly promising for such groups, as these methods can handle different foods and food cultures, without the need to be adapted to specific populations like some other methods (i.e. FFQ), and they require low-moderate levels of participant burden and literacy [6, 7]. Several such image-based methods have been developed that utilise different types of devices, such as personal digital assistants, handheld cameras, wearable cameras or (smart)phones [6, 7, 10], but many require apps, software, special equipment and/or training, even those that have successfully been tested and used in resource-poor areas [12, 13]. In addition, dietary assessment methods in general are not routinely validated in such populations [10, 14].

We wanted to develop a method that, while being as simple as possible, could be used to gather valid data in a future intervention study with circa 300 parent-child dyads from areas of socioeconomic deprivation. The intervention is aimed at supporting parents of 6-year-old children in their efforts to help their child develop healthy habits and is described elsewhere [15]. Many of the target parents do not speak Swedish proficiently and the family may not eat according to traditional Swedish dietary patterns. The intervention aims to improve dietary intake “at home”, i.e. all time when the child is not at school, and so the method needed to be able to capture this. In Sweden, all children are provided with lunch at pre-school and school, and no food is brought from home. The lunch is generally of good quality, freshly prepared and no sweet or fried foods are served, and so it is food eaten outside of school where there is often most room for improvement.

The purpose of this study was therefore to determine if photos taken by parents using just a camera-equipped mobile phone could provide valid information on selected dietary intake for 5- to 7-year olds relative to another method, multiple 24-h recalls (24HRs). We also investigated the effect of including an end-of-day review question. A second aim was to evaluate the relative validity of an alternative method, an adapted version of a previously-validated FFQ, compared to multiple 24HRs. A third aim was to explore the parents’ experiences with the photo method, supplemented by data from the research staff who interacted with them. The hypothesis was that the photo method would have reasonable validity, be superior to the FFQ, and be feasible and acceptable even for this target group.

## Methods

### Study design, participants and recruitment

As we wanted to evaluate the relative validity of the photo method quantitatively and also identify important hindering and facilitating contextual factors influencing its use, we used a mixed-methods design [16]. We recruited parents who were participants of a previous intervention conducted several years previously [17]. They were eligible if they had a child aged between five and seven years. As this dietary assessment method was developed specifically for use in families with low socioeconomic position (SEP), a further criterium was that families lived in specific areas in Stockholm County with low levels of employment and education, that have been identified by the government as being in need of socio-economic development. As compensation, we offered a pair of tickets to a children’s activity centre. For the qualitative evaluation, persons collecting the quantitative data (see below) were also included as participants.

### Dietary assessment overview

In summary, three quantitative methods were used: parents took photos using the photo method (PM) on three days (of which one was a weekend day), provided a 24HR to a dietitian after each of the three days, and completed an FFQ the following week, regarding intake the week of the PM and 24HRs. The 24HRs are considered the reference method for the purpose of this study.

The primary outcome of interest was the weekly intake of selected foods and drinks, outside of school hours only. These food items, chosen as being of relevance for energy balance and hereafter referred to as “indicator foods”, were “fruits and vegetables” (including legumes), “energy dense sweet/salty foods” (cakes/biscuits, sweets/chocolate, ice-cream, crisps/savoury snacks) and “sweet drinks” (soft drinks including sugar-free versions, sweetened milk and fruit juice). All intakes were assessed in terms of volume, not weight. Volume was possible to assess from photographs, intakes were reported in the 24HR as household units, and the FFQ also asked about volumes. This eliminated the need for conversion factors to weight which can be an additional source of error [18]. To enable comparisons between all three methods, all intakes were converted to dl (1 dl, ≈0.42 US cups [19]) and extrapolated to intakes per week.

### Assessment via phone camera

This was a form of ecological momentary assessment (EMA) where parents took photos in real-time and in the real-life setting whenever an eating event occurred [9]. Parents took photos on three days; two weekdays (Fridays excluded) and one weekend day. As we were monitoring feasibility, half were assigned non-consecutive weekdays, half consecutive weekdays. Although we were interested in selected foods and drinks, parents were simply told to take photos of *all* food and drinks. This was to avoid having to give very detailed instructions of which exact foods to include, which would be complicated, would be leading (by alerting them to the foods we were interested in, e.g. “you only need to take a photo if vegetables are present”) and would introduce error by relying on parents’ knowledge of foods. They were to take photos, including any additional portions, before and after each eating occasion, using their phone’s camera. Only consumption outside of school hours was to be recorded, but regardless of whether it was at home, at a relative’s house or sports club etc. If parents were not present, another caregiver could send photos to us, or parents could tell us via SMS (text message) afterwards. Parents were instructed to send the photos by MMS (multimedia message) or e-mail straight away, or, if necessary, later the same day.

No training was provided to parents; instructions were sent by post 2 weeks prior to data collection. Instructions were developed for parents with a low proficiency in Swedish; they were brief, with easy-to read text, with photos illustrating good and bad examples. The main points were that the photo should not be blurry; if the item was difficult to identify (e.g. juice) the parent should provide a brief description in the text message; and if a food was served in a package (e.g. a bag of sweets) the parents should specify how much had been consumed if it was not obvious from the photo. Leftovers were to be photographed/reported, even if there were none. A credit card-sized fiducial marker with cm markings and a coloured grid was provided to later help coders better estimate portion size and colour. The card was to be included in all photos, but in order to keep the method as simple as possible, no detailed instructions about the composition of the photo (i.e. angles, distances, lighting etc.) were given, and parents were not provided with any other equipment, such as standardised tableware or measuring cups.

Interaction with parents was minimal in order to lessen both researcher and participant burden. A reminder was sent by pre-scheduled text message the night before and the morning of each photo recording day. Research staff reviewed the photos in near real-time and contacted the parent by text message only if necessary, e.g. if a photo was blurry or to ask for detail about amounts when a post-meal photo was missing. If no photos had been received, the parent was reminded again or a new day for photo recording was scheduled. Parents were asked to reschedule if the child was sick on the appointed day.

As this was an event-contingent dietary EMA [9] we found it difficult to be sure when a parent was finished reporting for the day. Although we wanted to keep contact to a minimum, we decided to introduce an extra question in the evening: “Have you photographed all food and drinks your child consumed before and after school today? If no, please specify what food/drinks have not been photographed and how much was consumed”. This decision to deviate from the protocol was a pragmatic one, partly in order to know when a parent was finished, partly to serve as a prompt for commonly forgotten foods. Because the data collection had begun and was staggered, and parents were recording on different days, some parents got this question on only one of their days, some on two, and many on three. This enabled us to additionally compare differences between days with and without this review question.

**Fig. 1:**
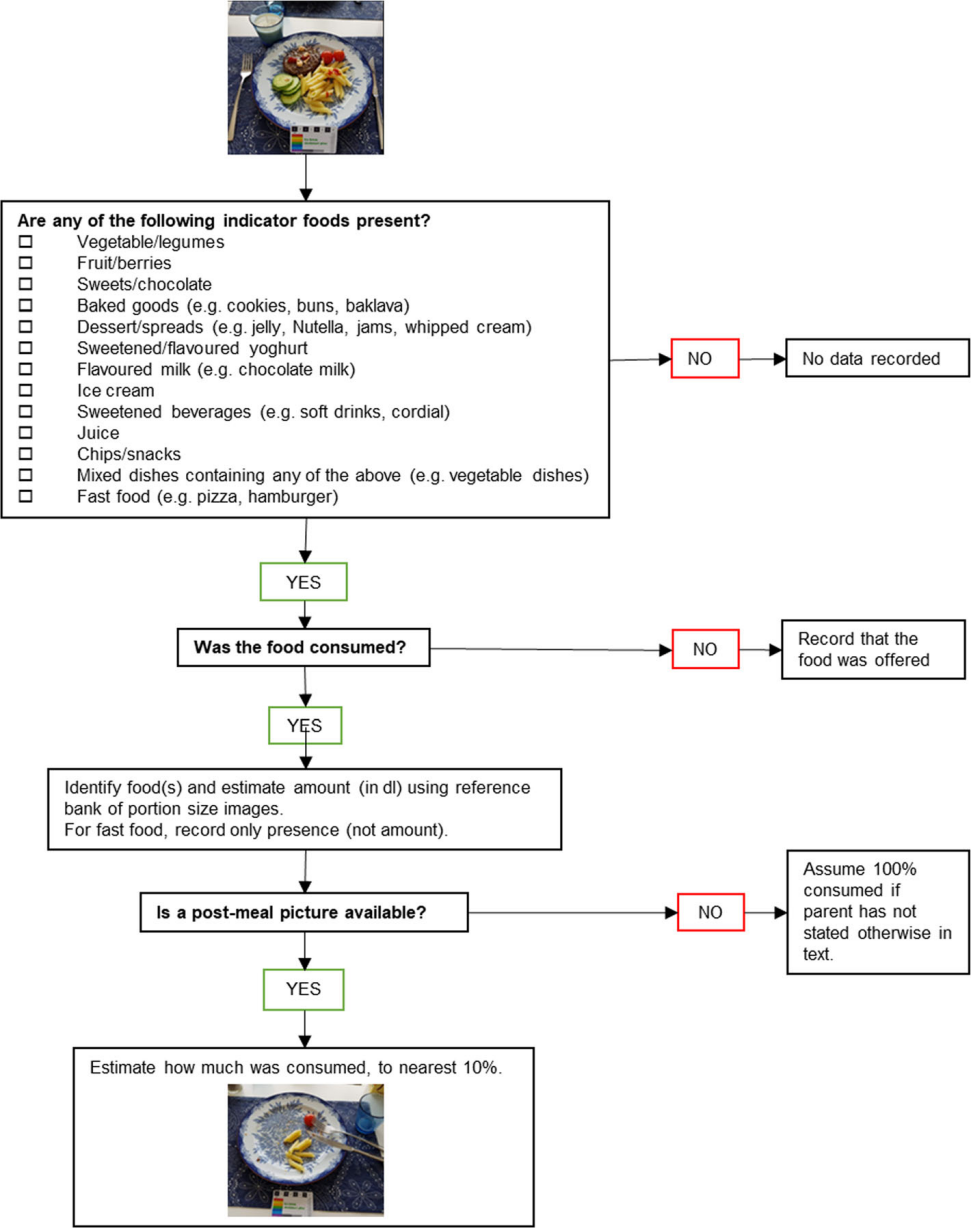
Protocol for coding of photos

The coding of the photos was performed manually. Two nutritionists (one a nutrition masters student; the other qualified and employed as a research assistant) coded independently, following a standard operational procedure which they helped to develop. The photos were assessed to determine 1) the presence or absence of indicator foods/drinks 2) the volume of indicator foods/drinks offered and 3) the percentage of the indicator food/drink that was consumed (Fig. 1). If a parent forgot to take a photo and only reported food/drink consumption by text, this was indicated in the protocol and the amount of food/drink reported was recorded. To standardise the assessment by the coders, a list of commonly consumed indicator foods/drinks was generated, and a library of reference photographs were created showing what one dl of these looked like in different states (e.g. sliced, chopped, whole). A list of standardised portions was also created. These were used for drinks served in glasses or cups, as these volumes were particularly difficult to estimate, or when the volume of the food was unclear (e.g. due to poor photo quality). Mixed dishes (e.g. stews, soups) were assessed using standardised portions and the volume of indicator foods was calculated based on standard recipes from the National Food Agency [20]. All photos were coded by both coders and the average of the two was used. The coders demonstrated high to very high levels of agreement, with single measure inter-class correlations (ICC, plus 95% CI) for fruits and vegetables, energy dense foods and sweet drinks of 0.985 (0.963–0.994), 0.891 (0.740–0.957) and 0.987 (0.966–0.995), respectively.

### Repeated 24-h recalls

The day after each of the three PM days, parents completed a 24HR by telephone with a licenced dietitian. Parents were assigned to one of two dietitians, both of whom had clinical experience of conducting dietary recalls with parents of young children. The dietitian asked the parent to recall all the food and drinks the child had consumed the previous day, without either of them consulting the photographs. They used a structured protocol, following the multiple-pass method [21], as far as was practical. Amounts of food and drinks were reported in volume or units (e.g. whole fruits, biscuits). At the end of each interview, the parent was asked if the day could be considered a typical day, and if the parent was aware of having changed their behaviour because of the need to photograph. To harmonise the dietitians’ technique, both dietitians first performed a test interview with a parent of a child in the targeted age group, but who was not part of the study. The test interviews were recorded and listened to by EP and ÅN, and instructions to the dietitians were clarified where necessary. The 24HR protocols were reviewed by one of the two nutritionists who coded the photos, but after a wash-out period of at least 4 weeks between coding tasks. The indicator foods were extracted from the protocols and, where necessary, volumes recorded in household units were converted to dl.

### Food-frequency questionnaires

Parents completed a web-based questionnaire measuring dietary intake 1 week after completion of the photo recording and 24HRs. For this, a revised version of the Eating and Physical Activity Questionnaire (EPAQ) was used. The EPAQ is a semi-quantitative FFQ measuring servings of the child’s intake of the same nine indicator foods mentioned above, in the home environment [22]. The original EPAQ measured intake during the previous day and was validated against one 24HR with parents of 2- to 5-year-old children in Australia. We adapted it to cover the previous week and therefore wanted to validate it against multiple 24HRs. To assist parents with a low proficiency in Swedish, easy-to read instructions and examples of servings both in text and pictures were also included.

### Qualitative data

To evaluate the parents’ experiences of barriers and facilitators related to using the photo method, at the end of data collection parents completed a web-based survey with open-ended questions reflecting barriers and facilitators. The questions posed were: What was your overall impression of the method? What was easy or good about using the photo method? What was difficult or less good about using the photo method? Was there any occasion when you/the parents found it difficult to take photos (yes/no)? If yes, describe the situation. What could make it easier for you/the parents to use the photo method? The research assistant who coordinated all data collection (KK) also completed the survey. In addition, KK kept a field diary with comments and reflections about barriers and facilitators to using the photo method and this data was also included. Semi-structured interviews were also conducted by EP and ÅN with both dietitians in order to capture what they perceived as barriers and facilitators regarding the photo method in this target group.

### Quantitative data analysis

For the purpose of this study, multiple 24HRs is considered the reference method. Three, or even two, 24HRs have been shown to be valid for the assessment of energy in this age group at group level [23–25]. Intakes assessed by both PM and FFQ were therefore compared to those assessed by 24HR. As this is a study of relative validity, all comparisons and results are relative, i.e. under-reported is to be read as “relatively under-reported”. To enable comparison between methods, all intakes were converted to dl per week. For 24HR- and PM-data, intakes on weekdays were weighted by 2.5 and on weekend days by 2. Sensitivity analyses performed without this weighting did not result in appreciable differences.

The evaluation of relative validity is based on the results of several tests: Spearman’s rho was calculated to assess the strength of associations and a Wilcoxon signed-rank test was used to test for differences in median intakes. Differences were plotted against the mean of the methods to create a Bland-Altman plot, which illustrates the mean bias, the 95% CI of the bias and any trend in the bias, i.e. if the size of the difference was not fixed relative to the mean. The study, when participants with incomplete data were excluded (*n* = 2), was sufficiently powered, with an alpha of 0.05 and beta of 0.8, to detect correlation coefficients of 0.600 or higher [26]. Analysis was performed using IBM SPSS Statistics v25 [27].

### Qualitative data analysis

Qualitative manifest analysis was applied to the data [28]. Each respondent comprised a unit of analysis. Questionnaires and the field diary were already in text format; interviews were transcribed verbatim by ÅN. All text was read repeatedly to gain familiarity with data. Meaning units relevant for the research question were first marked in text, and then given a code corresponding to the research question of barriers and facilitators to using the photo-method. Codes were kept close to the text. To identify sub-categories and categories, we first searched for and identified patterns of similarity among codes, and merged codes into sub-categories in accordance with the identified patterns. In the next step, we searched for and identified patterns of similarity among sub-categories and merged sub-categories into categories. In order to reflect over the strength of different barriers and facilitators [29], the number of participants expressing a certain sub-category of barrier and facilitator was counted. This was made possible by keeping both codes and sub-categories manifest and close to the text. Frequencies are displayed in a group format, parents and data collectors, as the two groups have different roles in the study, and in order to ensure the concealment of participant identity. Analysis was conducted by ÅN and reviewed by EP to ensure credibility of findings. ÅN and EP were not in contact with the parents at any point. Quotations for each category are included to further ensure credibility.

## Results

Of the 21 families recruited, one did not submit the food photos in time and another failed to complete a questionnaire, resulting in a final study population of 19 parent-child dyads (90.5% completion rate) for the quantitative portion. Parents of 11 boys and 8 girls participated, and the average age of the children was 6.3 years (SD 0.8). The parent that provided the dietary information was the mother in 14 cases (74%). Approximately a third of responding parents had less than 12 years of education (37%, *n* = 7), and the majority were born outside of Sweden, either in Europe (11%, *n* = 2) or outside of Europe (63%, *n* = 12). Almost half (47%, *n* = 9) were not proficient in Swedish.

### Differences between methods

**Fig. 2.**
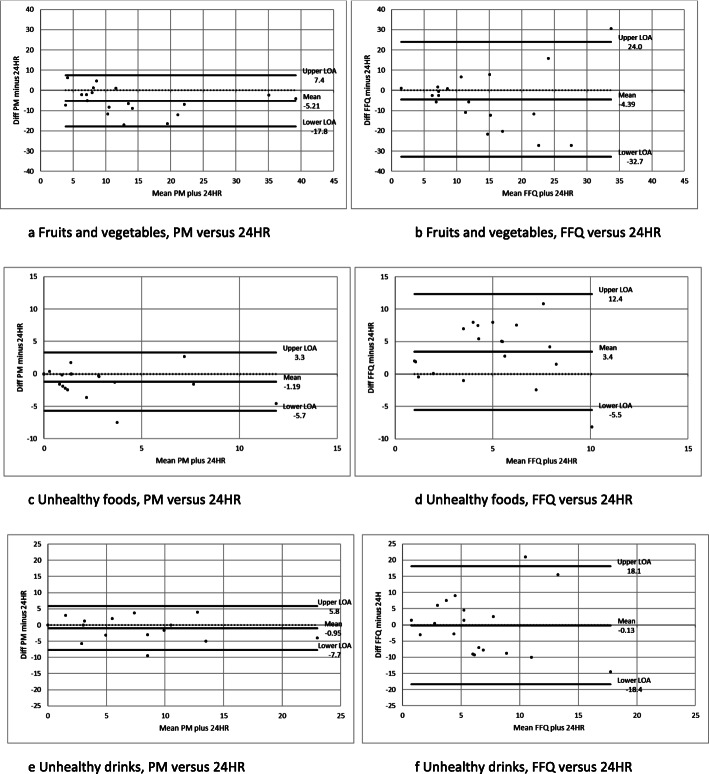
**a**-**f** Bland-Altman plots illustrating the differences in intakes of food categories assessed by photo method (PM) and FFQ. Differences are plotted against the mean of intakes assessed by 24HR plus PM and FFQ, respectively. Upper and lower levels of agreement (LOA) are mean+/− 2SD. Axes have been chosen so that results are comparable between methods. All units are dl/week

The intake per week according to the two methods – PM and FFQ – and in relation to the 24HRs (reference) are shown in Table 1. The absolute intakes according to the PM were highly correlated with those of the 24HRs for all categories. However, the PM underestimated the absolute intakes of all but one of the food categories (Table 1). For fruits and vegetables, approximately a third was not captured by PM, corresponding to 5.6 dl/w. The Bland-Altman plots (Fig. 2) illustrate the underestimation: mean difference is below zero for all food categories. The limits of agreement (mean+/−2SD) were wide: 25.3, 9.0 and 13.5 dl/w for fruits and vegetables, energy dense foods and sweet drinks respectively. This indicates large differences between methods at an individual level. However, the correlation between mean intakes and differences was low (not shown, all with *p* > 0.05), and the plots suggest the underestimation by photo assessment is fairly consistent across levels of intake.

**Table 1:** Intakes (dl/week) according to the three methods used

	24 h recall (reference)^1^		Photo method^1^								FFQ						
Category	Mean intake (dl)	Cons. (%)	Mean intake (dl)	Diff. (%)	Wilcoxon signed rank test (P)		Spearman’s rho		Cons. (%)	Correctly classified (%)	Mean intake (dl)	Diff. (%)	Wilcoxon signed rank test (P)		Spearman’s rho	Cons. (%)	Correctly classified (%)
Fruits and vegetables	16.8 (10.4)	100	11.2 (9.5)	−33	0.003	**	0.609	**	100	100	12.1 (11.3)	−28	0.126		0.342	100	100
- fruit	7.8 (6.2)	84	4.8 (6.0)	−38	0.020	*	0.562	*	68	81	6.2 (5.8)	−21	0.372		0.386	100	100
- vegetables	9.0 (6.4)	100	6.4 (4.5)	−29	0.006	**	0.688	**	100	100	5.9 (7.5)	−35	0.022	*	0.377	100	100
Energy dense foods	3.0 (3.7)	79	2.0 (3.0)	−32	0.056		0.612	**	58	73	7.3 (3.5)	144	0.004	**	0.106	100	100
- cakes/biscuits	1.4 (2.0)	63	0.8 (1.6)	−43	0.074		0.575	*	32	50	1.6 (1.3)	13	0.280		0.485	84	92
- sweets/chocolate	0.4 (0.7)	37	0.3 (0.8)	−14	0.866		0.600	**	21	57	2.0 (1.8)	405	0.001	**	0.420	68	86
- ice-cream	0.7 (1.7)	21	0.7 (1.7)	−2	1.000		0.997	**	21	100	1.6 (1.1)	132	0.021	*	−0.202	84	75
- crisps/savoury snacks	0.5 (1.3)	16	0.2 (0.6)	−56	0.285		0.990	**	16	100	2.2 (1.7)	318	0.007	**	−0.026	79	67
Sweet drinks	6.9 (6.5)	74	5.7 (5.7)	−17	0.147		0.845	**	74	93	6.6 (5.9)	−5	0.383		0.259	95	100
- soft drinks	3.0 (3.5)	58	1.9 (3.2)	−35	0.074		0.593	**	37	55	2.0 (3.4)	−33	0.333		−0.315	53	46
- sweetened milk	1.5 (2.9)	26	1.2 (2.5)	−19	0.066		0.998	**	26	100	3.0 (4.2)	102	0.195		0.275	63	100
- fruit juice	2.4 (4.9)	37	2.6 (4.3)	8	0.573		0.761	**	53	86	1.6 (2.4)	−35	0.900		−0,261	53	43

^1^ Intakes on weekdays and weekend day weighted to estimate weekly intake

Intakes are described using mean (std.dev) but are often skewed due to zero intakes, so non-parametric statistical tests are performed

Correctly classified means correctly classified as consumers

*N* = 19 with complete data from all 3 methods

Cons = consumers

Diff = difference in mean intakes

* *P* < 0.05, ** *P* < 0.01

The FFQ both over- and underestimated the absolute intakes in relation to the 24HRs and intakes were significantly correlated for only one sub-category (Table 1). Fruits and vegetable categories/sub-categories were underreported (the difference for vegetables was significant), all unhealthy categories/sub-categories were over-reported (most differences significant) and sweet drinks categories/sub-categories were both over- and underreported (no differences significant). The limits of agreement were roughly twice as wide as for the PM: 56.7, 17.9 and 36.5 dl/w, for fruits and vegetables, energy dense foods and sweet drinks, respectively. Furthermore, Bland-Altman plots (Fig. 2) suggest increasing variability with increasing mean intake.

### Differences according to whether review question was posed

Of the 57 measurement days in total (19 × 3), this question was not asked on 18 days, it was asked but not answered on 20, and it was answered on 19 days. Although the groups were not strictly independent statistically speaking, when compared to the 24HRs, correlations for all categories were generally larger, and under-reporting less pronounced, when a review question was posed and when an answer was received (Table 2).

**Table 2:** Differences in intakes reported by photo method across all measurement days (*n* = 57 total) according to review question status

			Spearman’s rho^1^						Mean intakes (dl/d)								
	All days		Answer received		No answer		No question		Answer received			No answer			No question		
Category	*n* = 57		*n* = 19		*n* = 20		*n* = 18		Recall	Photo	Diff. (%)	Recall	Photo	Diff. (%)	Recall	Photo	Diff. (%)
Fruit and vegetables	0.655	**	0.853	**	0.593	**	0.437		2.92	2.18	−25	2.39	1.30	−46	1.95	1.37	−30
Energy dense foods	0.742	**	0.684	**	0.605	**	0.792	**	0.49	0.52	6	0.36	0.07	−80	0.53	0.38	−28
Sweet drinks	0.812	**	0.846	**	0.921	**	0.651	**	1.21	1.03	−15	0.88	0.71	−19	0.99	0.80	−19

^1^ Correlation between intakes measured by photo method and intakes measured by 24HR

Answer received = a review question was sent in the evening, and an answer was received. No answer = a review question was sent but no answer was received. No question = a review question was not sent on that day

Diff. = Difference

** *P* < 0.01

### Photo method feasibility and acceptability

Regarding the overall perception of the PM, eleven (58%) of the parents expressed that their overall impression of the PM was that it was “easy”, whereas no parent expressed the overall impression of the method as being “difficult”. In addition, eleven of the 19 parents responded “no” to the question: “Was there any occasion when you found it difficult to take photos?”

### Barriers to using the photo method

#### Demanding

Within the category related to demanding aspects of using the PM, identified sub-categories included the method being time-consuming, difficulties in remembering to take the photos and to do it correctly, and difficulties in following instructions (Table 3).

**Table 3:** Number of parents and data collectors expressing each of the barriers and facilitators, plus quotes

Barriers			Facilitators		
**Category** Sub-category	**Parents**	**Data collectors**	**Category** Sub-category	**Parents**	**Data collectors**
**Demanding**			**Uncomplicated**		
Time-consuming	M8, M10, M11, M14	DC1, DC3	Easy way of expressing oneself	M5, M9, M11, M13	DC1, DC2
*“It was stressful to have to take photos of the food during the three days […] Mornings are stressful as it is and then to have to photograph the breakfast. That was a stressful moment that I didn’t appreciate” M8* *“I guess it [was] hard for them to find time for it. As I understood it they had several children or other tasks” DC3*			*“That you don’t have to explain so much.” M13* *“Often fast response to prompts that we sent* via *text message.” DC1*		
Having to remember and do correctly	F1, M8, M10, M11, M13, M14, M16	DC1, DC2, DC3	User-friendly instructions and reminders	M3, M5, M10, M14	DC1, DC2
*“Sometimes hard to remember that you were to photograph everything [… and] to make it a good photo where the food was displayed clearly.” M16* *“Some parents forgot the “after” picture […] Difficult to remember to photograph ALL food” DC1*			*“Good and informative instructions, easy-to use reference card” M14* *“Parents remembered additional things that had been eaten when we sent the evening reminders” DC1*		
Difficulty in understanding instructions		DC1, DC2	Simple method	F2, M5, M6, M8, M9, M15, M16	DC1, DC2
*“I think that some hadn’t understood that they were supposed to include everything. It feels like some thought that it was only, like, the meals. What you would think of as food, maybe you wouldn’t think that sweets are food, you know.” DC2*			*“That you could send it as an MMS, simple and easy.” M6* *“Everyone can take photos.” F2* *“Felt like the technology of taking photos and sending by MMS was easy for most of them, and that they got reminder the day before and the same day.” DC1*		
**Challenging in irregular situations**			**Engaging**		
Differences in family meal preference	M5, M7, M14	DC1, DC3	Child involvement	M3, M4, M7, M12, F5	DC2, DC3
*“It’s trickier when you have a child that naturally eats what they feel like from the breakfast table, first one thing, then another thing, then a third thing in more of a buffet style.” M14* *“They don’t all [in all the families] have a standard of eating together, but you take a little [food] here and a little there, and then to take pictures of every piece you know…, maybe it wasn’t put on a plate like [for example] sauce and potatoes, they had a different way of eating than what might be considered as standard.” DC3*			*“Nice that children also enjoy participating in the photographing.” F5* *“And the children thought it was fun with the photos. So they were pretty enthusiastic.” DC2*		
Intake away from home	M1, M10, M15	DC1, DC2, DC3	Positive experience	M1, M2, M7, M10, F4, M15	
*“When we went on an excursion and were going to bring other children.” M15* *“Snacks between meals, sweets, they ate at someone else’s or situations when you get a fruit in the shop, or when you are out at a festival or get candy floss or things like that.” DC2*			*“It was good and educational to participate.” M1*		
Parental absence	M6, M10, M13, M14	DC1, DC2, DC3	Well-planned meals and intake at home	M1, M4, M10, M11	
*“The child was at granny’s.” M13* *“Children who go and take [food and drink] a lot by themselves. They go and pick [food] from the fridge and things like that.” DC2*			*“Before the meal you had to think of everything, food, drink, toppings, ketchup* etc.*” M4*		

*F* father, *M* mother, *DC* data collector

Time-consuming: Some parents experienced the PM as more time-consuming than they first imagined. Taking photos each time the child ate or drank something during 3 days was a source of stress as parents juggled other chores, childcare etc.

Having to remember and do things correctly: Parents described it as demanding at times to take good, clear photos, fit all food and drinks in one photo, take photos of everything the child ate/drank both before and after the child had finished. In addition to feeling under pressure to take photos of good quality, parents also found it demanding to remember to take the photos and to keep the fiducial card with them at all times. The data collectors (KK and dietitians) too described that parents forgetting to take photos constituted a barrier to the method. In addition, one of the data collectors expressed that having to be available to monitor/review parents’ photos or text messages late at night constituted a barrier.

Difficulties in following instructions: The data collectors described difficulties the parents had with understanding the instructions for taking photos, either due to a lack of proficiency in Swedish, or simply not understanding that photos should be taken of all dietary intake, not only at regular meals.

#### Challenging in irregular situations

Within the category related to challenging aspects of using the PM in irregular situations, the identified sub-categories involved differences in family meal preferences, food intake outside of the home, and parental absence.

Differences in family meal preferences: Parents described difficulties in taking photos due to different preferences for eating styles or structures for serving food in the family, such as when the child had already started eating, when the child wanted a refill of a certain food while other foods that had already been photographed were left on the plate, or when the child preferred to have food little by little on the plate, buffet-style. Data collectors agreed, and in addition mentioned other difficulties with parental reporting in situations such as when food was readily available at all times and children could help themselves, e.g. a bowl of nuts on the living room table, as well as difficulty in identifying foods that were unfamiliar to them, i.e. from other food cultures.

Intake outside of home: Both parents and data collectors expressed that being away from home constituted a barrier to taking photos, e.g. during weekend activities, on excursions, or when shopping. In these situations it was easy for the parent to forget to take photos, or to forget the fiducial card. When someone else, e.g. a friend of the child, was present it was also more awkward to take photos.

Parental absence: Both parents and data collectors expressed that the absence of a parent engaged in the study constituted a barrier to taking photos. The child could be with another adult, e.g. a grandmother, or at a friend's house, or the child could eat or drink at home without the parent knowing. In addition, if both parents in the family were not equally engaged in the study, spending time with the non-engaged parent would constitute a barrier.

### Facilitators to using the photo method

#### Uncomplicated

Within the category related to uncomplicated aspects of using the PM, identified sub-categories involved the method being an easy way of expressing oneself, instructions and reminders being user-friendly, and the method being simple to use.

Easy way of expressing oneself: Parents described how the structure of taking photos was an easy way of conveying a message, not having to explain very much, that photos can convey more than words, and that the researcher could see for themselves via the photos. Also, by sending photos, parents had the opportunity to report culturally specific foods different from the Swedish ones that would otherwise be difficult to describe. Data collectors described how parents would send short explanations and description via text message without prompts, that clarifications were seldom needed, and that photos were often clear.

User-friendly instructions and reminders: Parents described the instructions as informative, the examples as clear, and the fiducial card as user-friendly. Data collectors described the evening reminders as useful; parents would remember occasions where they had forgotten to take photos and would respond with that information.

Simple method: Parents described the method of taking photos complemented by text messages via their phones as simple. Parents described how taking photos is something everyone can do, that using the phone is an everyday habit. They found it very simple to send photos via MMS and to simply explain additional information via text messages. The data collectors agreed on the simplicity of the method as a facilitator.

#### Engaging

Within the category related to engaging aspects of using the photo-method the identified sub-categories involved child involvement, positive experiences, and planned meals and food intake at home.

Child involvement: Both parents and data collectors described how children were happy, engaged, and found it fun to have photos taken of their food and drinks. In addition, both parents and data collectors described how some children would remind parents to take photos. Some parents found it positive that the child seemed to be more aware of what they were eating and drinking.

Positive experience: Parents mentioned the positive experience of using the method as being a facilitator. The reasons for this differed: some found it fun, some found it a novelty, educating, or interesting to see the amount of food and drinks recorded.

Well-planned meals and intake at home: Parents expressed that thorough planning of meals facilitated taking photos. In addition, being at home simplified the photographing procedure.

## Discussion

In this study of relative validity, we found that assessment of selected foods via camera-equipped mobile phones resulted in under-reporting of both healthy and less healthy foods, and sometimes the differences were large enough to be of what could be termed “clinical significance”. However, the under-reporting was fairly consistent and did not vary according to level of intake. This suggests that comparing intakes at group level as well as classifying children as high or low consumers based on ranking may be possible with this simple method. The moderate relative validity of the photo method, combined with the acceptability of the photo method means that, for the purposes of assessing group intakes in a difficult-to-reach population group of parents with low SEP and with cultural diversity, we consider the PM feasible. It performed better than the FFQ, which resulted in both under-reporting and over-reporting compared to 24HRs: correlations were lower, limits of agreements in Bland-Altman plots were wider, and ranking within the group was likely to be unreliable.

Although the use of several tests to assess relative validity is common, it is not always easy to summarise multiple results clearly [30]. Basing conclusions solely on Bland-Altman limits of agreement are often problematic, due to the difficulty in defining “acceptable” differences [30]. Our conclusions regarding relative validity are therefore not based on the result of any individual test, but of the results when taken as a whole. As only relative validity was assessed, information on absolute intake is unclear and results using this method may not be generalizable externally. As with all methods that perform better at group level than at individual level, the risk of misclassification will be present.

A review of mobile phone-based methods (although not all photo-based) found that all methods showed similar, but not superior, validity or reliability when compared with conventional methods, validated against energy or nutrient intakes [10]. A similar mobile phone-based method, TECH [23], found that selected foods, similar to those in our study, could be estimated accurately compared to multiple 24HRs. Rank order correlations were comparable to ours (Spearman’s r 0.665–0.896) but in that study no significant differences in median intakes were seen [23]. Importantly, that study also measured total energy expenditure objectively using doubly labelled water and found that energy intakes reported via TECH did not differ significantly from total energy expenditure. That study measured 4 days of intake and was carried out in a population of 39 Swedish children and their parents. The parents were well-educated and motivated, and were given more detailed instructions for photographing and provided with tableware. We found somewhat similar results in a different group of parents with a simpler method, although the trade-off is presumably less accurate measurement of total energy (not an outcome of interest for our study) and a risk of under-reporting of certain foods. This is particularly likely at the level of the individual, as indicated by the wide limits of agreement in the Bland-Altman plots, something commonly seen in dietary validation studies, particularly smaller ones [31].

The overall impression from the majority of the parents was that the PM was “easy”, which is in line with a previous research. Several reviews have found that participants’ satisfaction and preferences for mobile phone dietary assessment methods were higher than those for conventional methods [5–7, 10]. The most frequently mentioned facilitator was that the method and technology was simple, easy to use and in line with family everyday life where “everyone” has a phone, can take photos and is used to doing so. Other facilitators were that the method was an easy way of expressing oneself: parents appreciated not having to explain much, and the method allowed for conveying e.g. culture-specific foods. These are distinct advantages over methods such as FFQs which should ideally be adjusted for each context and population [1]. Data collectors also found that parents responded quickly to text message questions and reminders. Parents expressed that the instructions were easy to understand, and data collectors also found that the evening review question led to parents remembering foods they had not photographed during the day, which was also borne out by the quantitative results.

The most frequently occurring barrier was that parents forgot to take photos. This was tackled by introducing an additional reminder at the end of each measurement day. The reminder often resulted in additional data and thus constitutes one way of handling the barrier, and many EMA protocols include such an end-of-day survey [9]. Future studies could ask participants prior to data collection if they want reminders at set time intervals, or specific times, and parents could also have the option to ask for more frequent reminders during data collection, which could be pre-scheduled to decrease burden on data collectors. Other barriers had to do with parents not being present, the child being away from home or situations when the child helped themselves to food. These were also voiced as potential barriers in the study by McCloskey et al. in a similar population [32]. These barriers could be tackled by providing parents with strategies for the specific situations, e.g. providing them with instructions for their specific family or helping them to instruct a caregiver. In addition, instructions could be further simplified with additional pictures and/or videos. Studies that collect data in this way for longer than three days should bear in mind that more or other barriers may emerge.

A major advantage of using simple phone photography means it is not necessary to devote resources to developing an app, getting participants to register and install it, and requiring installation on potentially multiple caregivers’ phones. Any camera-equipped phone with data-transfer capability can be used, and the process is familiar to participants. Whether sending MMS incurs costs for participants will of course vary depending on data plans and countries. Participants can wait until they have access to (free) wifi to send photos, or researchers could offer to make credit available. Once the MMS are stored by the receiving phone, researchers have the flexibility to develop a back-up and storage system that is appropriate for them and their regulatory environment. This requires planning however, as a large amount of data is quickly created, which must be sorted efficiently [8, 9]. We developed one solution, of several possible, for this (information available on request). While much research is ongoing to achieve automatic identification of foods from images, it remains a long way off in practice [33]. Manual coding is very time-consuming, but does shift the burden of summarizing and estimating intakes, a non-trivial source of error, from parents to researchers. However, evaluating the contents of photos is still challenging for researchers, especially when mixed dishes are involved or if total energy is of interest [34]. We limited the foods of interest to a small number and closely monitored inter-rater reliability.

This was a small study, limited by the pool of participants we had access to from another study, and so results of individual analyses, and in particular sub-group analyses, are to be interpreted with caution. Even though we had hoped to recruit more participants with lower education, a high proportion were non-Swedish born and many were not proficient in Swedish. A review of mobile-phone based methods found that they were commonly validated in populations not representative of the general population, very often women and from academic, even nutrition, settings [10], and a more recent review of EMAs found that few had validated their protocols at all [9].

We evaluated only relative validity against another method, using three 24HRs as a reference [35]. Although not a gold-standard, 24HRs are commonly used as reference methods [36] and three [25], or as few as two [24], 24HRs have been shown to be valid for the assessment of energy in this age group at group level. As 24HRs were the reference method, we interpreted differences seen as due to under-reporting by the PM rather than over-reporting by the 24HRs. Although possible that parents provided wrong information due to memory problems, lack of knowledge, desirability bias (wanting or not to mention foods perceived to lead to judgment) or poor technique from the interviewer, in general under-reporting is more common than over-reporting with this method [37, 38]. While the evaluation of absolute validity against an objective recovery method, of e.g. total energy expenditure by doubly-labelled water, would have been superior, we were neither interested in measuring the total day’s intake, nor in energy per se. Direct observation is another potential objective method, but because we were interested in food consumed everywhere *other* than a fixed environment (i.e. when not at school) this was not feasible either.

When only examining relative validity researchers should at least compare methods that vary in some important characteristics in order to minimise correlated errors [35]. We therefore compared the prospective, self-reported PM, and the retrospective, self-reported FFQ, to retrospective, interview-mediated 24HRs. There are different schools of thought as to whether the reference method should be administered first or not [35, 39]. We led with the PM which meant that parents were completing 24HRs for days they had been actively taking photos on, which may explain the higher correlations seen between these two methods. We did not provide instructions regarding the angle at which photos should be taken, in order to simplify the information. The vast majority of the photos were taken at an overhead angle, but the estimations were possibly more uncertain for the ones that were not, and instructions could be included in future versions.

Over-reporting via the FFQ, which was particularly noticeable for some energy dense foods, could instead be a sign that this population had difficulty with an FFQ, which is cognitively challenging. It has also been suggested that parents perhaps think of food as served rather than consumed when completing FFQs and do not take leftovers into consideration [40]. It could also be that intakes via 24HRs were being under-reported, despite others finding them valid in this age-group. Common reasons for under-reporting include social desirability bias and reactivity, i.e. altered behaviour. Dietitians conducting the interviews were vigilant for the former and specifically asked questions to capture the later. The dietitians reported that they did not get the sense that parents were consciously reluctant to report unhealthy intakes, and reported that once mentioned, they were forthcoming with amounts and details (data not shown). No parent gave an indication of altered behaviour (data not shown).

The open-ended questions forming the base of the qualitative data only allows for manifest analyses and thus, only provides limited in-depth information. Semi-structured interviews with parents, which would have provided richer data, were deemed too burdensome for the parents in this study. To ensure credibility of findings, triangulation of data (survey, interviews with dietitians, and field diary) were used in the study. In addition, the audit trail described in the methods section, illustrative quotes, and intersubjective agreement in the analysis process vouch for trustworthiness of the qualitative findings.

## Conclusions

The use of simple camera-equipped mobile phones by parents to assess the intake of selected food and drinks of young children was an acceptable and feasible method for these parents, many of whom had a low socioeconomic position and/or were not proficient in the local language. The simplicity of this method compared to some other image-based assessment tools makes this approach attractive for researchers with limited resources. While there is a need for simplified methods and minimizing participant burden, too few reminders and review questions may result in poorer data quality. Both the quantitative and qualitative results suggest relative under-reporting was an issue. Further work is required to be certain of the method’s absolute validity and usefulness for accurately measuring intake at individual level. The relative validity of the method for measuring intake at group level was moderate, and was better than another method evaluated simultaneously.

## Data Availability

The datasets used and/or analysed during the current study are available from the corresponding author on reasonable request.

## Abbreviations

24HR: 24-h recalls
FFQ: food frequency questionnaire
PM: photo method
SMS: text message (short message service)
MMS: multimedia message service
EMA: ecological momentary assessment
